# Supplementary material for: The Staphylococcus aureus-antagonizing human nasal commensal Staphylococcus lugdunensis depends on siderophore piracy
Source: Microbiome. 2024 Oct 22;12:213. doi: 10.1186/s40168-024-01913-x (PMC11495082; doi:10.1186/s40168-024-01913-x)
Supplement: Supplementary file 2 — Supplementary Material 1: Supplementary Table S1. Comparison of metagenome data with community state types (CST). Relative proportions (in percent) of selected taxa that had been described as signature for community state types (CST) as described in [11]. Most abundant taxa are highlighted in bold. Round 1 or 2 indicates the corresponding time of metagenome analysis after 18 months or 23 months, respectively. CST: classification of designated CST types according to the prevalence of indicator taxa in the microbiomes or none if not determinable (nd). CST6 for sample H-2 was based on a relative proportion of 54.4% Moraxella (not listed). For S. lugdunensis, the corresponding relative DNA abundances are also shown (in percent). The culture-based carrier states determined in parallel to the metagenome sequencing are indicated under “Culture”: Slu, S. lugdunensis carrier; Sau, S. aureus carrier; co, co-carrier; non, non-carrier. Supplementary Table S2. Correlation coefficients of species present in the nasal microbiomes. Correlation coefficients were inferred based on a centered log-ratio-transformed abundance table of species reads by correlation network analyses using the Namap/Pearson algorithm of MetagenoNets [20] with thresholds for prevalence and occurrence of 0.1% and 20%, respectively. The table shows the resulting correlation measures for 28 species throughout all 24 samples. Positive correlations are emphasized by green shades (with intensity corresponding to the hight of the values), while negative correlations are shaded in red. Supplementary Table S3. Bacterial strains used in this study. Supplementary Table S4. Plasmids and oligonucleotides used in this study. [file 40168_2024_1913_MOESM1_ESM.pdf]

Supplemental Data.

Suppl. Table. S1. Comparison of metagenome data with community state types (CST). Relative proportions (in percent) of selected taxa that had been described as signature for community state types (CST) as described in [11]. Most abundant taxa are highlighted in bold. Round 1 or 2 indicates the corresponding time of metagenome analysis after 18 months or 23 months, respectively. CST: classification of designated CST types according to the prevalence of indicator taxa in the microbiomes or none if not determinable (nd). CST6 for sample H-2 was based on a relative proportion of 54.4 % *Moraxella* (not listed). For *S. lugdunensis,* the corresponding relative DNA abundances are also shown (in percent). The culture-based carrier states determined in parallel to the metagenome sequencing are indicated under "Culture": Slu, *S. lugdunensis* carrier; Sau, *S. aureus* carrier; co, co-carrier; non, non-carrier.

Sample	Round	*Corynebacterium*	*S. epidermidis*	*S. aureus*	*Dolosigranulum*	CST	*S. lugdunensis*	Culture
**A**	1	**67.2**	21.6	3.7	0.0	**3/5**	2.11	Slu
	2	12.4	**69.3**	4.7	0.0	**3/5**	0.77	Slu
**B**	1	**60.4**	14.3	0.9	0.0	**3/5**	0.49	Slu
	2	**44.3**	37.7	1.4	0.0	**3/5**	0.27	Slu
**C**	1	11.2	0.2	0.2	**83.3**	**7**	0.03	Slu
	2	12.0	0.3	0.1	**82.9**	**7**	0.19	Slu
**D**	1	3.6	7.3	6.2	0.0	nd	0.00	non
	2	4.4	32.0	1.4	0.0	nd	0.07	non
**E**	1	44.1	**51.3**	2.1	0.0	**3/5**	0.12	non
	2	23.1	**71.5**	3.2	0.0	**3/5**	0.20	non
**F**	1	24.8	1.6	27.0	0.0	nd	0.19	co
	2	9.1	26.5	2.0	0.1	nd	0.39	co
**G**	1	39.8	7.3	6.1	0.0	nd	0.00	Slu
	2	**45.6**	**46.7**	1.3	0.0	**3/5**	3.37	Slu
**H**	1	**92.9**	2.0	1.2	0.0	**3/5**	0.00	non
	2	3.9	0.2	1.8	23.0	**6**	0.00	non
**I**	1	3.0	0.1	0.3	**91.2**	**7**	0.00	non
	2	8.2	0.1	1.2	**73.7**	**7**	0.00	non
**J**	1	18.6	2.2	0.8	**68.6**	**7**	0.00	non
	2	9.3	3.1	0.4	**73.3**	**7**	0.00	non
**K**	1	1.8	0.5	**91.4**	0.0	**1**	0.04	Sau
	2	**46.5**	1.3	**67.5**	0.1	**1**	0.27	co
**L**	1	**61.2**	44.6	1.2	0.0	**3/5**	0.08	non
	2	26.0	**73.2**	2.8	0.0	**3/5**	0.11	non

Suppl. Table S2. Correlation coefficients of species present in the nasal microbiomes. Correlation coefficients were inferred based on a centered log-ratio-transformed abundance table of species reads by correlation network analyses using the Namap/Pearson algorithm of MetagenoNets [20] with thresholds for prevalence and occurrence of 0.1% and 20%, respectively. The table shows the resulting correlation measures for 28 species throughout all 24 samples. Positive correlations are emphasized by green shades (with intensity corresponding to the hight of the values), while negative correlations are shaded in red.

	Anaerococcus sp.	*Campylobacter jejuni*	*Chlamydia sp.*	*Corynebacterium accolens*	*Corynebacterium aurimucosum*	*Corynebacterium casei*	*Corynebacterium diphtheriae*	*Corynebacterium efficiens*	*Corynebacterium propinquum*	*Corynebacterium simulans*	*Corynebacterium striatum*	*Corynebacterium tuberculostearicum*	*Cutibacterium acnes*	*Dolosigranulum pigrum*	*Finegoldia magna*	*Lawsonella clevelandensis*	*Listeria monocytogenes*	*Mycobacterium sp.*	*Neisseria sp.*	*Rhodococcus sp.*	*Salmonella enterica*	*Staphylococcus aureus*	*Staphylococcus capitis*	*Staphylococcus epidermidis*	*Staphylococcus lugdunensis*	*Streptococcus anginosus*	*Streptococcus pneumoniae*	*Streptomyces sp.*
Anaerococcus sp.	1.00	0.26	-0.14	0.14	0.47	0.08	0.19	0.24	-0.26	0.07	0.18	0.56	0.62	-0.16	0.27	0.24	0.23	-0.08	0.20	0.28	0.20	0.41	0.27	0.66	0.05	0.19	-0.54	0.25
Campylobacter jejuni	0.26	1.00	0.43	-0.02	0.42	0.09	0.27	-0.09	0.14	0.08	0.18	0.46	0.29	0.15	0.08	0.05	0.67	0.23	0.96	0.15	0.93	0.26	-0.09	0.15	-0.02	0.17	-0.03	0.14
Chlamydia sp.	-0.14	0.43	1.00	-0.34	-0.21	-0.36	-0.23	-0.19	0.13	0.12	-0.15	-0.05	-0.21	-0.01	-0.05	0.09	0.32	0.43	0.42	0.01	0.42	0.06	-0.27	-0.28	0.01	0.00	0.36	0.13
Corynebacterium accolens	0.14	-0.02	-0.34	1.00	0.60	0.32	0.66	0.27	-0.36	0.43	0.74	0.54	0.56	-0.04	0.15	0.51	0.15	0.02	-0.16	0.50	-0.12	0.07	0.20	0.59	0.32	0.52	-0.28	0.40
Corynebacterium aurimucosum	0.47	0.42	-0.21	0.60	1.00	0.52	0.83	0.21	-0.25	0.59	0.79	0.85	0.76	-0.14	0.19	0.52	0.40	0.17	0.34	0.57	0.37	0.46	0.37	0.59	0.15	0.56	-0.30	0.46
Corynebacterium casei	0.08	0.09	-0.36	0.32	0.52	1.00	0.49	0.18	0.19	0.40	0.42	0.36	0.27	0.15	0.28	0.15	-0.07	-0.14	0.16	0.08	0.15	0.16	0.56	0.38	0.34	0.05	-0.38	-0.03
Corynebacterium diphtheriae	0.19	0.27	-0.23	0.66	0.83	0.49	1.00	0.28	-0.22	0.67	0.90	0.64	0.72	-0.06	0.23	0.63	0.44	0.13	0.16	0.77	0.14	0.32	0.33	0.58	0.24	0.73	-0.24	0.64
Corynebacterium efficiens	0.24	-0.09	-0.19	0.27	0.21	0.18	0.28	1.00	-0.27	0.26	0.42	0.19	0.33	0.01	0.34	0.06	0.05	-0.06	-0.14	0.64	-0.22	-0.06	0.46	0.32	0.35	0.59	-0.37	0.51
Corynebacterium propinquum	-0.26	0.14	0.13	-0.36	-0.25	0.19	-0.22	-0.27	1.00	0.05	-0.45	-0.20	-0.22	0.66	-0.13	-0.30	0.11	-0.01	0.23	-0.32	0.28	-0.16	-0.07	-0.20	-0.01	-0.31	0.28	-0.30
Corynebacterium simulans	0.07	0.08	0.12	0.43	0.59	0.40	0.67	0.26	0.05	1.00	0.66	0.46	0.50	0.13	0.04	0.45	0.35	0.53	0.00	0.67	0.08	0.31	0.41	0.31	0.28	0.65	-0.01	0.65
Corynebacterium striatum	0.18	0.18	-0.15	0.74	0.79	0.42	0.90	0.42	-0.45	0.66	1.00	0.62	0.66	-0.24	0.32	0.60	0.34	0.22	0.06	0.76	0.05	0.27	0.33	0.49	0.35	0.78	-0.23	0.67
Corynebacterium tuberculostearicum	0.56	0.46	-0.05	0.54	0.85	0.36	0.64	0.19	-0.20	0.46	0.62	1.00	0.86	-0.17	0.28	0.50	0.33	0.11	0.36	0.49	0.35	0.26	0.25	0.65	0.41	0.50	-0.49	0.46
Cutibacterium acnes	0.62	0.29	-0.21	0.56	0.76	0.27	0.72	0.33	-0.22	0.50	0.66	0.86	1.00	-0.11	0.27	0.52	0.35	0.07	0.14	0.66	0.13	0.27	0.28	0.74	0.38	0.62	-0.54	0.59
Dolosigranulum pigrum	-0.16	0.15	-0.01	-0.04	-0.14	0.15	-0.06	0.01	0.66	0.13	-0.24	-0.17	-0.11	1.00	-0.16	-0.41	0.19	-0.01	0.13	-0.07	0.19	-0.41	-0.07	-0.08	-0.03	-0.17	0.15	-0.01
Finegoldia magna	0.27	0.08	-0.05	0.15	0.19	0.28	0.23	0.34	-0.13	0.04	0.32	0.28	0.27	-0.16	1.00	0.33	-0.18	-0.38	0.07	0.22	-0.08	-0.10	-0.10	0.28	0.50	0.20	-0.41	0.30
Lawsonella clevelandensis	0.24	0.05	0.09	0.51	0.52	0.15	0.63	0.06	-0.30	0.45	0.60	0.50	0.52	-0.41	0.33	1.00	0.17	-0.05	-0.04	0.56	-0.10	0.46	0.07	0.49	0.28	0.55	-0.18	0.50
Listeria monocytogenes	0.23	0.67	0.32	0.15	0.40	-0.07	0.44	0.05	0.11	0.35	0.34	0.33	0.35	0.19	-0.18	0.17	1.00	0.54	0.55	0.47	0.58	0.32	0.13	0.21	-0.11	0.44	0.25	0.42
Mycobacterium sp.	-0.08	0.23	0.43	0.02	0.17	-0.14	0.13	-0.06	-0.01	0.53	0.22	0.11	0.07	-0.01	-0.38	-0.05	0.54	1.00	0.15	0.21	0.26	0.30	0.16	-0.07	-0.05	0.25	0.39	0.31
Neisseria sp.	0.20	0.96	0.42	-0.16	0.34	0.16	0.16	-0.14	0.23	0.00	0.06	0.36	0.14	0.13	0.07	-0.04	0.55	0.15	1.00	0.01	0.97	0.27	-0.02	0.02	-0.11	0.02	-0.04	-0.04
Rhodococcus sp.	0.28	0.15	0.01	0.50	0.57	0.08	0.77	0.64	-0.32	0.67	0.76	0.49	0.66	-0.07	0.22	0.56	0.47	0.21	0.01	1.00	-0.03	0.23	0.34	0.49	0.23	0.93	-0.22	0.90
Salmonella enterica	0.20	0.93	0.42	-0.12	0.37	0.15	0.14	-0.22	0.28	0.08	0.05	0.35	0.13	0.19	-0.08	-0.10	0.58	0.26	0.97	-0.03	1.00	0.30	0.00	-0.01	-0.19	-0.01	0.05	-0.09
Staphylococcus aureus	0.41	0.26	0.06	0.07	0.46	0.16	0.32	-0.06	-0.16	0.31	0.27	0.26	0.27	-0.41	-0.10	0.46	0.32	0.30	0.27	0.23	0.30	1.00	0.40	0.34	-0.22	0.29	-0.10	0.11
Staphylococcus capitis	0.27	-0.09	-0.27	0.20	0.37	0.56	0.33	0.46	-0.07	0.41	0.33	0.25	0.28	-0.07	-0.10	0.07	0.13	0.16	-0.02	0.34	0.00	0.40	1.00	0.33	0.14	0.25	-0.36	0.10
Staphylococcus epidermidis	0.66	0.15	-0.28	0.59	0.59	0.38	0.58	0.32	-0.20	0.31	0.49	0.65	0.74	-0.08	0.28	0.49	0.21	-0.07	0.02	0.49	-0.01	0.34	0.33	1.00	0.31	0.40	-0.64	0.43
Staphylococcus lugdunensis	0.05	-0.02	0.01	0.32	0.15	0.34	0.24	0.35	-0.01	0.28	0.35	0.41	0.38	-0.03	0.50	0.28	-0.11	-0.05	-0.11	0.23	-0.19	-0.22	0.14	0.31	1.00	0.27	-0.34	0.26
Streptococcus anginosus	0.19	0.17	0.00	0.52	0.56	0.05	0.73	0.59	-0.31	0.65	0.78	0.50	0.62	-0.17	0.20	0.55	0.44	0.25	0.02	0.93	-0.01	0.29	0.25	0.40	0.27	1.00	-0.16	0.85
Streptococcus pneumoniae	-0.54	-0.03	0.36	-0.28	-0.30	-0.38	-0.24	-0.37	0.28	-0.01	-0.23	-0.49	-0.54	0.15	-0.41	-0.18	0.25	0.39	-0.04	-0.22	0.05	-0.10	-0.36	-0.64	-0.34	-0.16	1.00	-0.19
Streptomyces sp.	0.25	0.14	0.13	0.40	0.46	-0.03	0.64	0.51	-0.30	0.65	0.67	0.46	0.59	-0.01	0.30	0.50	0.42	0.31	-0.04	0.90	-0.09	0.11	0.10	0.43	0.26	0.85	-0.19	1.00

Suppl. Table. S3. Bacterial strains used in this study

Bacterial strain	Description	Source or reference
Corynebacterium accolens		
DSM 44279	origin unknown	DSMZ
63VAs_B8	human nasal isolate	[41]
83VAs_B5	human nasal isolate	[41]
KPL 1824	human nasal isolate	[47]
KPL 1855	human nasal isolate	[49]
KPL 1996	human nasal isolate	[49]
Corynebacterium aurimucosum		
10VPs_Sm8	human nasal isolate	[41]
48/2	lesional site isolate	AG Peschel strain collection
Corynebacterium kroppenstedtii		
82VAs_B6	human nasal isolate	[41]
Corynebacterium propinquum		
8VAs_B3	human nasal isolate	[41]
63VAs_B4	human nasal isolate	[41]
P7-31	human nasal isolate	this study
56/2	human nasal isolate	AG Peschel strain collection
57/2	human nasal isolate	AG Peschel strain collection
Corynebacterium pseudodiphtheriticum		
87VAs_B4	human nasal isolate	[41]
90VAs_B3	human nasal isolate	[41]
P1-29	human nasal isolate	this study
P2-34	human nasal isolate	this study
P6-3	human nasal isolate	this study
Corynebacterium simulans		
81MNs_B1	human nasal isolate	[41]
50MNs_Sm2	human nasal isolate	[41]
50VAs_B5	human nasal isolate	[41]
79/2	groin isolate	AG Peschel strain collection
73/2	groin isolate	AG Peschel strain collection
77/2	groin isolate	AG Peschel strain collection
Corynebacterium striatum		
24/2	human nasal isolate	AG Peschel strain collection
46/2	lesion site isolate	AG Peschel strain collection
38/2	groin isolate	AG Peschel strain collection
Corynebacterium tuberculostearicum		
87VAs_B5	human nasal isolate	[41]
12VAs_B4	human nasal isolate	[41]
Cutibacterium acnes		
44VAs_Sa4	human nasal isolate	[41]

50VAs_Sa1	human nasal isolate	[41]
83VAs_Sa3	human nasal isolate	[41]
87VAs_SaT9	human nasal isolate	[41]
Cutibacterium avidum		
Clone#11	human nasal isolate	AG Peschel strain collection
Dolosigranulum pigrum		
9VAs_B4	human nasal isolate	[41]
Escherichia coli		
DH5α	K-12 derivative	New England BioLabs
DC10B	Δ*dcm* in the DH10B background	[27]
CMFI_AI 1	axilla isolate	AG Peschel strain collection
CMFI_AI 2	groin isolate	AG Peschel strain collection
CMFI_AI 3	axilla isolate	AG Peschel strain collection
Finegoldia magna		
63 Vas_Sa4	human nasal isolate	[41]
83 Vas_Sa6	human nasal isolate	[41]
Klebsiella pneumoniae		
ATCC700603	human urine isolate	ATCC
Lawsonella clevelandensis		
DSM 45743	human nasal isolate	DSMZ
Moraxella catarrhalis		
44VAs_Sm4	human nasal isolate	[41]
80VAs_B4	human nasal isolate	[41]
Staphylococcus aureus		
USA300 LAC	CA-MRSA isolate	[50]
IVK41	human nasal isolate	[12]
IVK55	human nasal isolate	[12]
IVK56	human nasal isolate	[12]
IVK58	human nasal isolate	[12]
Staphylococcus capitis		
10VAs_KB2	human nasal isolate	[41]
44UNs_B2	human nasal isolate	[41]
50VAs_KB6	human nasal isolate	[41]
Staphylococcus caprae		
VA18305/14	human clinical isolate	Eppendorfklinikum, Hamburg
BK3880/14	human clinical isolate	Eppendorfklinikum, Hamburg
Staphylococcus epidermidis		
B1-1	human nasal isolate	this study
B1-5	human nasal isolate	this study
B1-10	human nasal isolate	this study
B5-7	human nasal isolate	this study
B5-7 Δ*sfaDAB*	deletion of *sfa* gene locus	this study
B5-26	human nasal isolate	this study
S1-2	human nasal isolate	this study

S1-15	human nasal isolate	this study
S1-27	human nasal isolate	this study
S2-2	human nasal isolate	this study
M14-19	human nasal isolate	this study
M15-6	human nasal isolate	this study
M16-32	human nasal isolate	this study
W1-7	human nasal isolate	this study
W1-7Δ*sfaDAB*	deletion of *sfa* gene locus	this study
W3-6	human nasal isolate	this study
W5-7	human nasal isolate	this study
W8-6	human nasal isolate	this study
Staphylococcus hominis		
50MNs_Sa6	human nasal isolate	[41]
89VPs_B7	human nasal isolate	[41]
9VPs_KB1	human nasal isolate	[41]
Staphylococcus lugdunensis		
HKU09-01	human skin infection isolate	[51]
HKU09-01 Δ*fhu*	deletion of fhu gene locus	this study
HKU09-01 Δ*fhu* pRB474_fhu	complementation of *fhu* gene locus; *fhu* genes encoded on pRB474	this study
IVK28	human nasal isolate	[12]
IVK28 Δ*fhu*	deletion of *fhu* gene locus	this study
Staphylococcus pettenkoferi		
210-70229632	human clinical isolate	AG Peschel strain collection
Mammaliicoccus sciuri		
DMS 16827		DSMZ
DMS 15613		DSMZ
9VPs_Sm2	human nasal isolate	[41]
Staphylococcus warneri		
VA16066/14	human clinical isolate	Eppendorfklinikum, Hamburg
BK6091/14	human clinical isolate	Eppendorfklinikum, Hamburg
Streptococcus pneumoniae		
ATCC 6301		ATCC
ATCC 49619		ATCC

Suppl. Table S4. Plasmids and oligonucleotides used in this study

Plasmid	Description	Reference
pIMAY	thermosensitive vector for allelic exchange	[27]
pRB474	*E. coli/S. aureus* shuttle vector; constitutive active expression vector	[52]

Oligonucleotides	Sequence (5'-3')	Purpose
(1) Fhu up for	TATAGGGCGAATTGGAGCTCTTTAGTATTT TTACCTTCTGCC	Generating upstream recombinant region for *fhu* deletion
(2) Fhu up rev	TAGAAAGTTGGGGAATTATGTAATAAAATT CGTGTTGGAA	generating upstream recombinant region for *fhu* deletion
(3) Fhu dw for	CATAATTCCCCAACTTTCTA	generating downstream recombinant region for *fhu* deletion
(4) Fhu dw rev	GGGAACAAAAGCTGGGTACCATTATTAAGC GTATTGATGATC	generating downstream recombinant region for *fhu* deletion
(5) fhu chrom. Up for	CTTTCACAGATGGACTCATT	Screening for chromosomal *fhu* deletion
(6) fhu chrom. Dw Rev	TATTAATCTCTGTATTTTTAGG	Screening for chromosomal *fhu* deletion
(7) fhu comp for	TTGCATGCCTGCAGGTCGACAAATAGAAAG TTGGGGAATT	generating *fhu* genes for complementation with pRB474
(8) fhu comp rev	GTACCCGGGGATCCTCTAGATTAAATAGAT TTAGCTTTATACAT	generating *fhu* genes for complementation with pRB474
(9) sfaDAB ko up for	CGAGGTCGACAATAGAGAGGGATAACTAAG	Generating upstream recombinant region for *sfaDAB* deletion
(10) sfaDAB ko up rev	TTCTAAATGATGGTCTATCATTTAGAACGT	generating upstream recombinant region for *sfaDAB* deletion
(11) sfaDAB ko dw for	TGATAGACCATCATTTAGAACAATGGATTT	generating downstream recombinant region for *sfaDAB* deletion
(12) sfaDAB ko dw rev	ATTGGAGCTCTTATAATTAGGTCCAAGATT	generating downstream recombinant region for *sfaDAB* deletion
(13) Chrom sfaDAB up for	TTATAAATCATTCTTTCTATGAA	Screening for chromosomal *sfaDAB* deletion

(14) Chrom sfaDAB dw rev	TTACTGGTGTGAAATGAATT	Screening for chromosomal *sfaDAB* deletion
(15) 473 Eco	CCTCAAGCTAGAGAGTCATTACCCC	Screening for pRB474_*fhu* positive transformants
(16) 473 Hind	CTGGATTTGTTCAGAACGCTCGG	Screening for pRB474_*fhu* positive transformants

Supplementary References

49. Tran TH, Roberts AQ, F. Escapa I, Gao W, Segre JA, Kong HH, et al. Metabolic capabilities are highly conserved among human nasal-associated Corynebacterium species in pangenomic analyses. bioRxiv. 2023:2023.06.05.543719; doi: 10.1101/2023.06.05.543719.
50. Diep BA, Otto M. The role of virulence determinants in community-associated MRSA pathogenesis. Trends Microbiol. 2008;16(8):361-9; doi: 10.1016/j.tim.2008.05.002.
51. Tse H, Tsoi HW, Leung SP, Lau SK, Woo PC, Yuen KY. Complete genome sequence of Staphylococcus lugdunensis strain HKU09-01. J Bacteriol. 2010;192(5):1471-2; doi: 10.1128/JB.01627-09.
52. Bruckner R. A series of shuttle vectors for Bacillus subtilis and Escherichia coli. Gene. 1992;122(1):187-92; doi: 10.1016/0378-1119(92)90048-t.
